# Plaque-Like CD34-Positive Dermal Fibroma of the Toe: A Case Report and Review of the Literature

**DOI:** 10.7759/cureus.85405

**Published:** 2025-06-05

**Authors:** Shaina Gagadam, Michael Shragher, Carmen Campanelli

**Affiliations:** 1 Dermatology, Philadelphia College of Osteopathic Medicine, Philadelphia, USA; 2 Dermatology, Temple University, Philadelphia, USA; 3 Dermatology, Yardley Dermatology Associates, Yardley, USA

**Keywords:** benign neoplasm, cd34+, dermal fibroma, medallion-like dermal dendrocyte hamartoma, mlddh, pdf, plaque-like cd34+ dermal fibroma, plaque-like dermal fibroma

## Abstract

Plaque-like CD34-positive dermal fibroma (PDF) is a rare, benign cutaneous neoplasm that is most commonly seen on the neck or trunk of young females. Diagnosis of PDF can be challenging due to its clinical heterogeneity and immunohistochemical overlap with other spindle cell neoplasms. We report the case of a 57-year-old female who presented with a painless, slowly enlarging pigmented macule on the dorsal aspect of her left first toe. Histopathologic analysis revealed a proliferation of wavy and spindle-shaped fibroblasts interwoven within the dermis, with immunohistochemistry showing strong CD34 positivity, consistent with a diagnosis of PDF. A comprehensive review of the literature was conducted to evaluate previously reported cases and anatomical distributions of PDFs. This review identified no prior reports of PDF involving the digits. To the best of our knowledge, this is the first documented case of a PDF occurring on the toe.

## Introduction

Plaque-like CD34-positive dermal fibroma (PDF), also referred to as medallion-like dermal dendrocyte hamartoma (MLDDH), is a rare, benign dermal neoplasm that typically presents as a slow-growing, asymptomatic plaque [1]. The pathogenesis of PDF remains poorly understood. Histologically, these lesions are characterized by the proliferation of spindle-shaped fibroblasts in the dermis, which are strongly positive for CD34 and negative for markers such as S100, Melan-A, and Factor XIIIa [1,2]. We present a case of a 57-year-old female with a PDF on the dorsal surface of her left first toe. This case demonstrates the potential for PDFs to occur in atypical sites, emphasizing the importance of thorough histopathologic and immunohistochemical evaluation for accurate diagnosis and appropriate management.

## Case presentation

**Figure 1:**
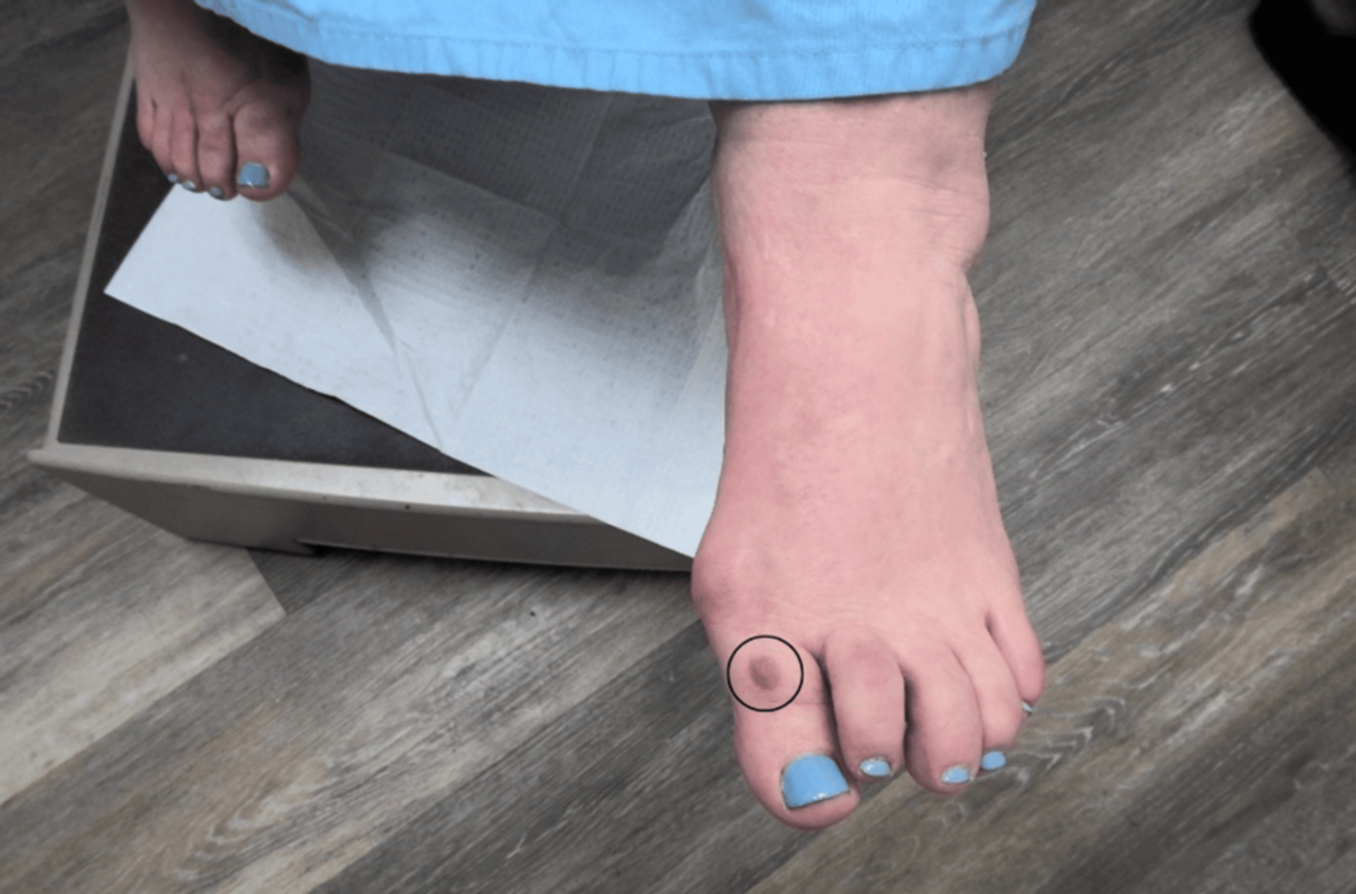
An irregularly pigmented macule located on the dorsal surface of the left great toe.

A 57-year-old female presented to the dermatology clinic for evaluation of a painless, suspicious lesion located on the dorsal surface of her left first toe. The lesion had been present for at least one year, but the patient noted a gradual increase in size and change in color over the three months prior to presentation. She denied any associated symptoms, trauma to the area, or recent changes in her overall health. Her medical history was significant for melanocytic nevi and basal cell carcinoma on the right lateral eyebrow, which was treated with Mohs micrographic surgery. The patient had no family history of similar dermatologic concerns and denied use of tanning beds, alcohol consumption, or tobacco use. On examination, the patient had an irregularly pigmented macule on the dorsal surface of the first left toe measuring approximately 8 mm (Figure 1).

**Figure 2:**
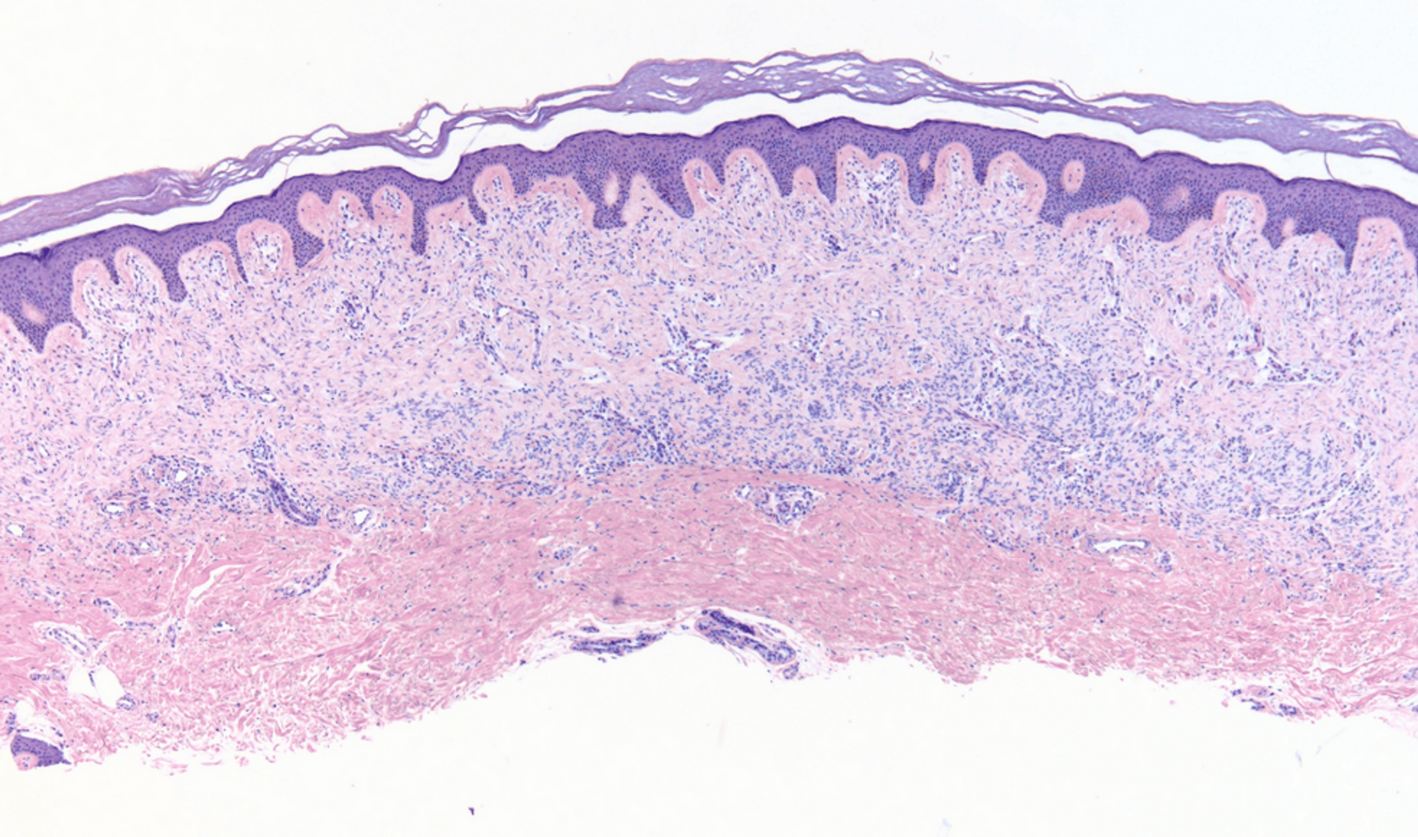
Histopathology showing a band-like proliferation of uniform spindle cells within the upper two-thirds of the dermis, with notable sparing of the superficial papillary dermis (hematoxylin and eosin, magnification ×4).

**Figure 3:**
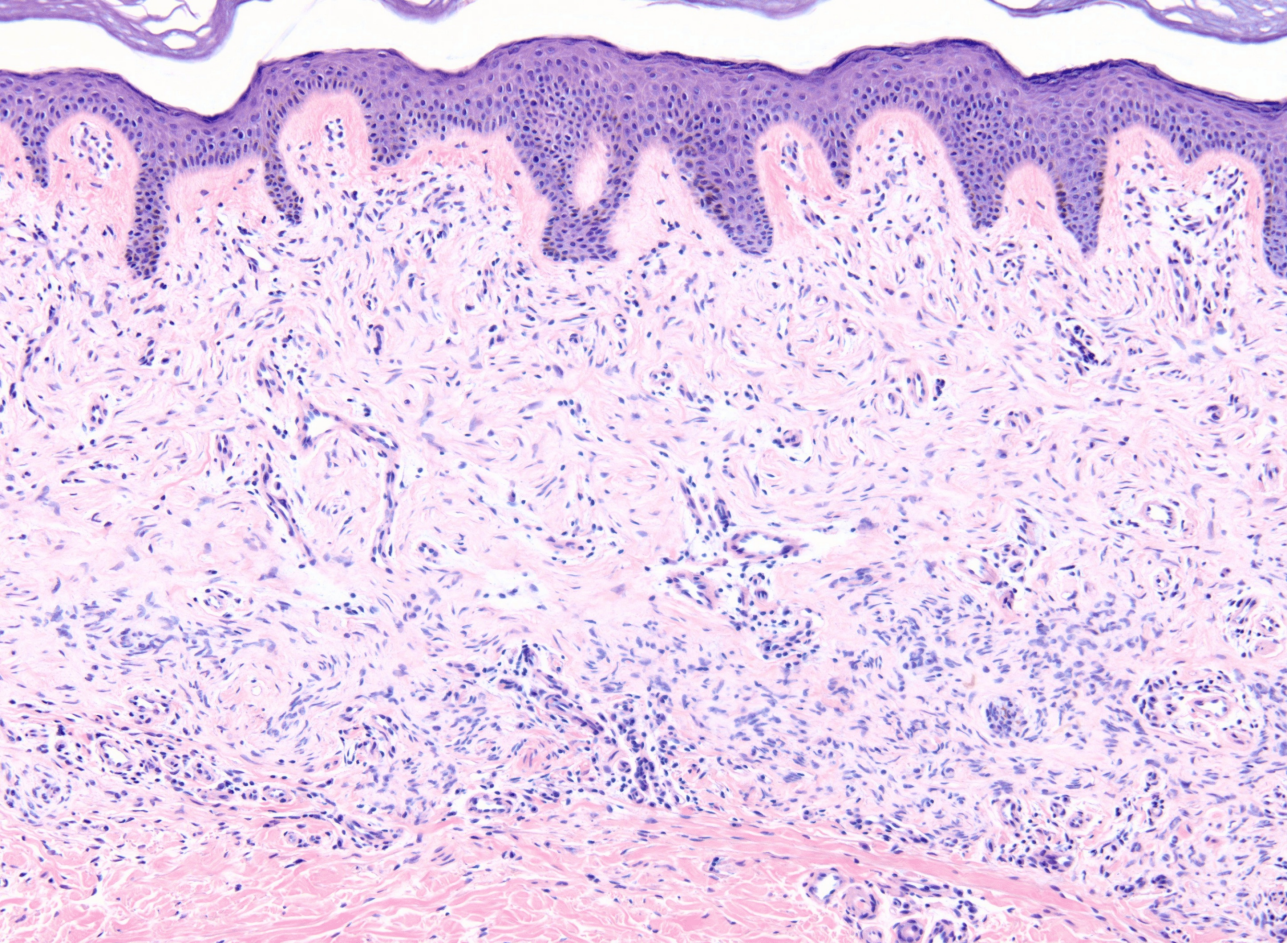
Within the upper reticular dermis is a proliferation of wavy and spindle cells oriented horizontally in the lower portion of the neoplasm and vertically in more superficial aspects (hematoxylin and eosin, magnification ×10).

A tangential biopsy was performed, and pathology revealed a neoplasm composed of wavy and spindle-shaped cells predominantly arranged in fascicles that interweave in the upper reticular dermis (Figure 2). The proliferation of cells exhibited a horizontal orientation in the lower portion of the neoplasm and a vertical orientation in the more superficial aspects (Figure 3).

**Figure 4:**
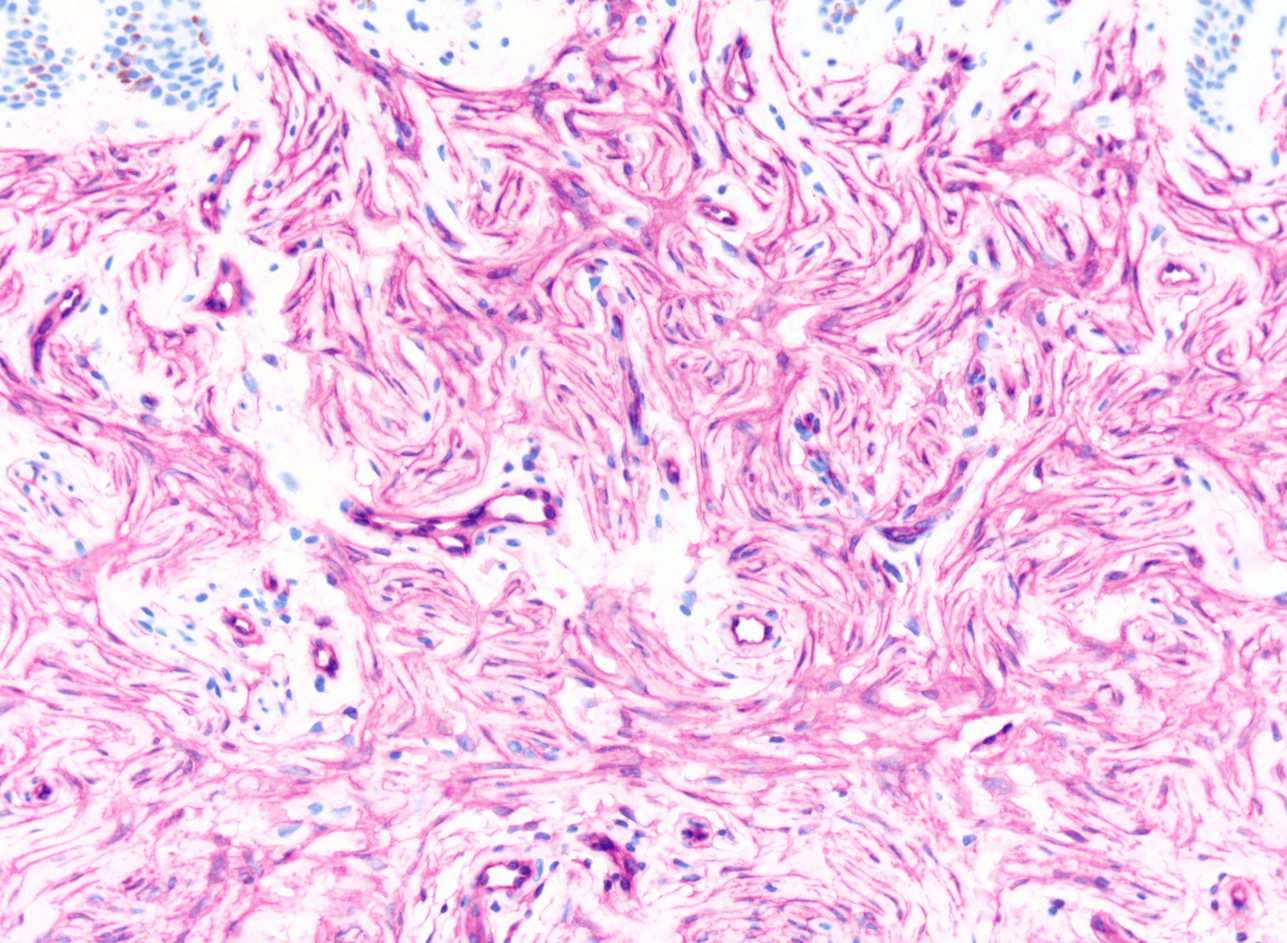
Diffuse CD34 positivity of spindle cells; CD34 immunostaining, magnification ×20.

In addition, immunohistochemistry staining was positive for CD34 and negative for Factor XIIIa, S100, and Melan-A (Figure 4). These features were consistent with the diagnosis of a PDF, also known as MLDDH.

**Figure 5:**
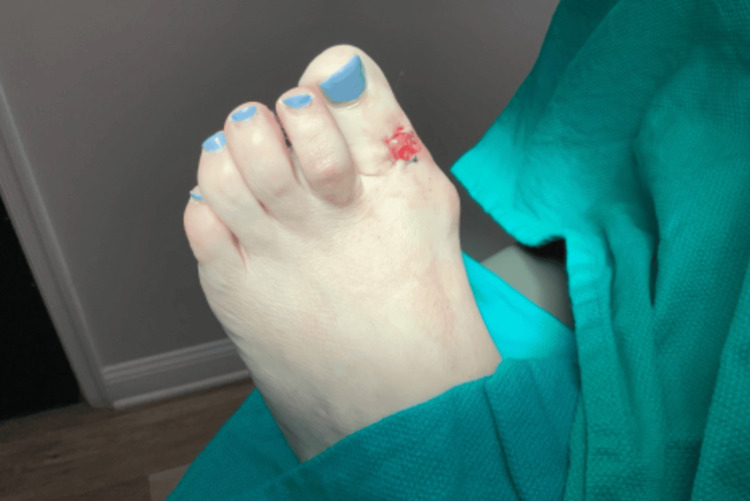
Intermediate purse-string closure following full-thickness excision.

The patient was then referred to the Mohs surgical office for further evaluation and management. Upon examination, an ulcer was identified at the site of the previous biopsy. A full-thickness excision was performed with 5 mm margins, and the specimen was submitted for pathology. The primary resection defect measured 14 x 11 x 4 mm, and the wound was closed using an intermediate purse-string technique (Figure 5). The procedure was well tolerated, and the patient was discharged the same day.

Pathology of the excised tissue confirmed the presence of an ulcer secondary to the prior biopsy, with granulation tissue observed beneath, but no residual neoplasm. At the 10-day postoperative follow-up, the patient reported redness and pain that began the day prior. Clinical examination revealed erythema and induration, although the lesion appeared to be healing well. Sutures were removed, and no recurrence of the lesion was noted. Gram stain and bacterial cultures were performed, both of which returned negative results. The patient was prescribed ciprofloxacin 500 mg BID and instructed to seek emergency care if any signs of infection progression occurred.

## Discussion

PDFs were first described by Rodriguez-Jurado et al. as well-defined, slightly atrophic, and asymptomatic congenital lesions in young female patients [1]. However, subsequent studies have revealed a broader spectrum of clinical presentations, including the presence of PDFs in non-congenital forms and in male patients, demonstrating the lesion’s clinical heterogeneity [3-5].

Although PDFs are often congenital, acquired variants have been reported, including cases such as ours and that of Mutgi et al., who described an acquired PDF on the posterior neck without epidermal alterations [3]. Additionally, Restano et al. reported a case of PDF in an 11-year-old boy, and Ducharme et al. documented a 36-year-old male with PDF, challenging the notion that these lesions predominantly affect female patients [4,5]. These findings demonstrate the diverse presentations of PDFs, emphasizing the importance of recognizing both congenital and acquired forms across all genders for accurate diagnosis and appropriate management.

The pathogenesis of PDFs remains poorly understood, despite their well-documented histologic and immunohistochemical features. A key diagnostic challenge lies in their immunohistochemical overlap with other fibroblastic neoplasms, particularly dermatofibrosarcoma protuberans (DFSP), as both entities stain positively for CD34 [2,6-8]. While they share similar staining patterns, PDFs can be distinguished from DFSPs based on their biological architecture and spindle cell morphology. In PDFs, there is a band-like proliferation of wavy and spindle-shaped cells arranged in short fascicles parallel to the epidermis, interwoven within the upper reticular dermis [1]. In contrast, DFSPs typically exhibit a storiform arrangement and are often more cellular and invasive, with a greater tendency to extend into the hypodermis and entangle fatty tissue [6,9].

A comprehensive review of the literature identifies a limited number of reported cases of PDFs, most of which are located on the neck or trunk, with few cases involving the proximal extremities [1-3,10]. Notably, there have been no reports of these lesions occurring on the digits. To our knowledge, this is the first documented case of a PDF occurring on the toe. This case contributes to our limited understanding of this rare entity by highlighting an unusual site of involvement and supporting its consideration in the diagnostic workup of cutaneous spindle cell neoplasms, particularly in atypical anatomic locations.

Treatment of PDFs depends on symptoms, lesion size, and location. Surgical management of PDF is most often guided by protocols established for DFSP, with complete excision and histologically clear margins considered the standard approach [11]. Of note, no universal guidelines exist regarding the optimal width of surgical margins [12]. Primary closure is typically sufficient, though larger defects may require reconstruction with local flaps or skin grafts [13]. Overall, while surgical excision is the primary treatment, management should be individualized based on clinical context, with long-term follow-up to monitor for recurrence. Further research is needed to establish treatment guidelines and better understand the molecular basis of PDFs.

## Conclusions

In conclusion, to the best of our knowledge, we present the first documented case of a PDF on the toe, an atypical location for this benign neoplasm. This case underscores the importance of recognizing the diverse presentations of PDFs and the essential role of histopathology and immunohistochemistry in confirming the diagnosis. Further research is needed to deepen our understanding of the pathogenesis of PDFs and their occurrence across different populations and anatomical sites.
